# Improved setup and positioning accuracy using a three‐point customized cushion/mask/bite‐block immobilization system for stereotactic reirradiation of head and neck cancer

**DOI:** 10.1120/jacmp.v17i3.6038

**Published:** 2016-05-08

**Authors:** He Wang, Congjun Wang, Samuel Tung, Andrew Wilson Dimmitt, Pei Fong Wong, Mark A. Edson, Adam S. Garden, David I. Rosenthal, Clifton D. Fuller, Gary B. Gunn, Vinita Takiar, Xin A. Wang, Dershan Luo, James N. Yang, Jennifer Wong, Jack Phan

**Affiliations:** ^1^ Department of Radiation Physics The University of Texas MD Anderson Cancer Center Houston TX USA; ^2^ Departments of Radiation Oncology The University of Texas MD Anderson Cancer Center Houston TX USA

**Keywords:** immobilization, head and neck cancer, stereotactic radiotherapy, ExacTrac, PTV margin

## Abstract

The purpose of this study was to investigate the setup and positioning uncertainty of a custom cushion/mask/bite‐block (CMB) immobilization system and determine PTV margin for image‐guided head and neck stereotactic ablative radiotherapy (HN‐SABR). We analyzed 105 treatment sessions among 21 patients treated with HN‐SABR for recurrent head and neck cancers using a custom CMB immobilization system. Initial patient setup was performed using the ExacTrac infrared (IR) tracking system and initial setup errors were based on comparison of ExacTrac IR tracking system to corrected online ExacTrac X‐rays images registered to treatment plans. Residual setup errors were determined using repeat verification X‐ray. The online ExacTrac corrections were compared to cone‐beam CT (CBCT) before treatment to assess agreement. Intrafractional positioning errors were determined using prebeam X‐rays. The systematic and random errors were analyzed. The initial translational setup errors were −0.8±1.3 mm, −0.8±1.6 mm, and 0.3±1.9 mm in AP, CC, and LR directions, respectively, with a three‐dimensional (3D) vector of 2.7±1.4 mm. The initial rotational errors were up to 2.4° if 6D couch is not available. CBCT agreed with ExacTrac X‐ray images to within 2 mm and 2.5°. The intrafractional uncertainties were 0.1±0.6 mm, 0.1±0.6 mm, and 0.2±0.5 mm in AP, CC, and LR directions, respectively, and $0.0^{\circ }\pm 0.5^{{}^{\circ}}$, $0.0^{\circ }\pm 0.6^{{}^{\circ}}$, and $-0.1^{\circ }\pm 0.4^{\circ }$ in yaw, roll, and pitch direction, respectively. The translational vector was 0.9±0.6 mm. The calculated PTV margins $m_{\text{PTV}(90,95)}$ were within 1.6 mm when using image guidance for online setup correction. The use of image guidance for online setup correction, in combination with our customized CMB device, highly restricted target motion during treatments and provided robust immobilization to ensure minimum dose of 95% to target volume with 2.0 mm PTV margin for HN‐SABR.

PACS number(s): 87.55.ne

## I. INTRODUCTION

Stereotactic ablative radiotherapy for the head and neck (HN‐SABR) is increasingly used for treatment of locally recurrent cancers.^(1)^ Modern linear accelerators (linacs) are appealing when equipped with advanced image guidance features and able to deliver fast and high‐quality treatments.^1^, ^2^ Essential considerations in linac‐based HN‐SABR include immobilization, image guidance, and real‐time motion tracking.

Recent advances in image guidance have allowed delivery of high‐dose radiation with increased confidence and safety in HN‐SABR. CBCT is available on most modern linacs and used for setup verification because of its capability to acquire volumetric images.^3^, ^4^, ^5^, ^6^ In‐room orthogonal X‐ray systems, such as the Brainlab ExacTrac X‐ray 6D system (Brainlab AG, Feldkirchen, Germany), are common add‐ons that allow for 6D setup verification and corrections prior to and during treatment.^7^, ^8^

While image guidance is normally used to correct initial setup errors, the robustness of the immobilization device is essential in maintaining the patient's position during the actual treatment (intrafractional) to constrain voluntary or involuntary motion. Several studies comparing post‐treatment to pretreatment imaging^6^, ^8^ have observed increasing setup errors in the post‐treatment images, indicating a potential for significant patient motion during treatment. These uncertainties need to be investigated to generate a confident planning target volume (PTV) margin to ensure the minimal expansion necessary to adequately cover targets while minimizing dose to nearby critical structures.

Besides being easy to implement and comfortable for the patients, a well‐developed immobilization system should be highly reproducible during the course of treatment and maximally limit patient motion. Compared to stereotactic head frame systems, frameless systems have the advantage of improved patient safety and comfort, with more convenient clinical and technical workflow. The inter‐ and intrafractional setup errors for head and neck patients immobilized with thermoplastic masks have been extensively investigated.^5^, ^9^, ^10^ Other immobilization devices, such as customizable head cushions and mouth pieces, can be coupled to the thermoplastic mask to further restrict patient motion.^(5)^

In this study, we report on the reproducibility and robustness of our unique customized frameless immobilization system utilizing a custom cushion, mask, and bite‐block (CMB) immobilization for HN‐SABR of recurrent cancers. ExacTrac X‐ray and CBCT imaging were combined to investigate inter‐ and intrafractional setup errors and the agreement between the two imaging modalities was assessed. Geometric uncertainties were analyzed to generate PTV margins from the target volume.

## II. MATERIALS AND METHODS

### A. Immobilization devices and SBRT procedure

**Figure 1:**
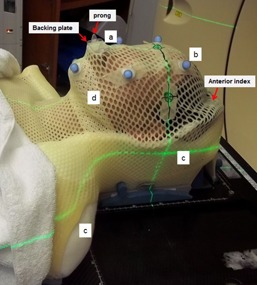
Immobilization of head‐and‐neck SBRT patients: (a) custom bite‐block conformed to patient's upper teeth, and indexed to the mask through two outward prongs and a small backing plate, limiting patient motion inside mask; (b) ExacTrac IR markers for in‐room alignment and motion tracking; (c) custom Klarity AccuCushion shaped to vertex and sides of the head, along the neck to shoulders; (d) custom Orfit head/neck and shoulder mask individualized to patient and indexed to the anterior of the custom cushion to create a flush fit and minimize mask air space.

We conducted an institutional review board–approved analysis of 21 patients in a total of 105 treatment sessions that underwent reirradiation with HN‐SABR from October 2013 through March 2015. Table 1 lists the patients and treatment information. We developed a CMB system by building on our existing thermoplastic mask immobilization used for conventionally fractionated IMRT. During simulation, patients were immobilized with a customized head and shoulder Klarity AccuCushion (Klarity Medical Products, Newark, OH), a thermoplastic head neck and shoulder mask (Orfit Industries America, Wijnegem, Belgium), and a customized bite‐block attachment fixated to the thermoplastic mask. The Klarity AccuCushion was shaped to the vertex and sides (mastoid process and temple) of the head, and along the curvature of trapezius and sternocleidomastoid muscles of the neck to shoulders. This created a posterior cup for indexing of the thermoplastic mask. A preheated moldable bite‐block (Precise Bite, Civico, Coralville, IA) was then conformed to the patient's upper teeth. The bite‐block contains two outward attachments that are indexed to the warm thermoplastic mask and secured by snapping a small backing plate to the mouth piece from outside the mask. Six IR passive reflection balls were placed on the mask before CT scans for ExacTrac use during treatment (Fig. 1).

**Table 1:** Information of HN‐SABR patients.

*Patient*	*Site*	*Prescription (Gy)*	*Fraction*	*Target volume (cm^3^)*	*Total MUs* ^a^	*Total Delivery Time* ^a^ *(min)*	*Group* ^b^
1	Right RP Node	45	5	7.37	1575	2.92	1
2	Right RP Node	45	5	9.95	1984	3.22	1
3	Right BOT	45	5	8.26	1566	2.67	2
4	Right OPX	45	5	15.27	2221	3.88	2
5	OPX	40	5	36.93	2819	4.74	1
6	Left RMT CA	40	5	59.82	2486	4.27	2
7	Right RP Node	45	5	8.98	2359	3.97	1
8	OPX	47.5	5	21.05	4259	7.13	1
9	Right BOT	45	5	17.19	2232	3.80	2
10	Skull Base	45	5	36.43	3388	6.79	1
11	Left NPX	45	5	21.56	4110	6.94	1
12	Right Neck	45	5	12.75	1875	3.17	2
13	Right Neck	45	5	24.59	2031	3.70	2
14	Post Pharynx	45	5	9.95	3000	5.15	1
15	Left RP Node	45	5	16.31	2018	3.37	1
16	Clivus	45	5	26.05	4505	7.52	1
17	Rt Neck	45	5	24.87	1885	3.16	2
18	C1‐C2	45	5	30.54	4365	7.05	2
19	Ethmoid Sinus	45	5	25.7	1649	2.72	1
20	Rt OPX	45	5	9.45	2285	3.87	2
21	BOT	45	5	29.18	1831	3.12	2

a Total MUs and total delivery time are per fraction.

b Group 1: skull base patients; Group 2:patients with targets inferior to C1 bone.

RP=retropharyngeal; BOT=base of tongue; OPX=oropharynx; RMT CA=retromolar trigone carcinoma.

For each patient, a simulation CT scan was acquired with 1 mm slice thickness and was coregistered in treatment planning system (Pinnacle, version 9.8, Philips Medical Systems, Andover, MA) with diagnostic MRI and/or PET‐CT, as well as treatment planning MRI images obtained with patient immobilized with the CMB. The target volume and critical structures were contoured, and a typical 2–3 mm PTV margin was added to the target volume by the attending physician. A median dose of 45 Gy (range 40 to 47.5 Gy) in 5 fractions prescribed to PTV was delivered every other day for an overall treatment time of two weeks. 6 MV photon beams on Varian TrueBeam STx system (Varian Medical Systems, Inc., Palo Alto, CA) with high definition (2.5 mm) leaflets were used for radiation delivery. All treatments were planned using 2‐3 arcs/beams volumetric‐modulated arc therapy (VMAT) technique to take advantage of efficient delivery and surrounding normal tissues sparing.

### B. Determining initial setup, residual, and intrafractional errors

#### B.1 Determining initial setup and residual errors

During each treatment, patients were comfortably immobilized into the CMB system. Each patient was initially positioned using the ExacTrac IR‐based optical system. Then two ExacTrac stereoscopic X‐rays were taken and fused to a set of fixed angle, digitally reconstructed radiographs generated from treatment planning CT. The best match was determined using translational variations in anterior–posterior (AP), craniocaudal (CC), and left–right (LR) directions, and rotational variations in yaw, roll, and pitch directions. Initial setup errors were determined from the difference in the translational (mm) and rotational (°) values between the ExacTrac IR‐based setup and ExacTrac X‐ray alignment. If the difference was more than 1 mm or 1°, shifts were applied to adjust the 6D robotic couch followed by repeat X‐ray images to verify the new position. The residual errors were calculated from the repeat verification ExacTrac X‐ray images.

#### B.2 Calculating ExacTrac X‐ray and CBCT agreement

ExacTrac X‐ray alignment is primarily based on bony anatomy on projection images, while CBCT alignment is based on target volume and surrounding tissue on volumetric CT images. Immediately after the ExacTrac X‐ray verification, a CBCT scan with 1 mm slice thickness was acquired, and volumetric matching of the target volume was performed between CBCT and planning CT on Varian 4D Integrated Treatment Console in both translational and rotation dimensions. This assessed the agreement between the volumetric CBCT alignment and the ExacTrac system. The fused images were then reviewed by onsite physician. If the difference was within 1 mm, no further shifts were made. If the difference was >1 mm, adjustments were made at the treating physician discretion. While ExacTrac X‐ray and CBCT differences were evaluated both translationally and rotationally, if a physician‐determined shift on CBCT was required, only translational shifts were allowed because the Brainlab 6D couch can only be controlled by one software system (which is utilized by the ExacTrac).

#### B.3 Calculating intrafractional motion

To assess intrafractional motion, ExacTrac X‐ray images were taken prior to each beam, and translational and rotational differences from treatment plan were measured. Since we utilize a 2–3 mm PTV margin, shifts were only applied if there was >2 mm discrepancy after taking into account the manual CBCT adjustment made by the physician. During the whole beam‐on time, the IR ball motion was monitored by therapist and physicist. The entire setup and delivery time for each treatment was typically 20–30 min.

### C. Analysis of positioning errors

For each patient, we calculated the mean and standard deviations (SD) of translational and rotational variations for the initial setup, residual setup errors, agreement of CBCT and ExacTrac X‐ray, and intrafractional uncertainties. To account for positional uncertainty of the cervical spine as shown in prior studies,^(11)^ results were also compared separately between skull base targets (Group 1, 11 patients, 55 sessions) and targets below C1 (Group 2, 10 patients, 50 sessions).

Systematic error $SM, random positioning errors σ, and PTV margin were also calculated for each step. In this study, $SM was defined as a standard deviation of average errors calculated for individual patients. σ was calculated as root‐mean‐square value of the observed random standard deviations for all patients involved.^(12)^ PTV margins m for confidence level p (percentage of patient population) to achieve dose level d (percentage of prescription dose) were determined using van Herk's formula:^(13)^

(1) $$m_{PTV(p,d)}=\alpha (p)\cdot \sum +\beta (d)\cdot \sigma$$

where *α* is a function of p, and *β* is a function of d. As an example, $m_{\text{PTV}(90,95)}$ is the minimal margin needed to achieve the confidence level of 90% of the patients received minimum dose of 95% to the target volume, and the suggested values are α=2.5, β=0.7. The 3D vector displacements were calculated as root sum of squares from the three translational dimensions.

### D. Statistical analysis

Statistical analyses for means and variances were performed using independent samples *t*‐test to determine significant differences of mean. The Levene's test was used to determine equity of variance. A *p* or *f* value <0.05 was considered to be statistically significant. All statistical analyses were performed using IBM SPSS Statistics 22.

## III. RESULTS

### A. Patient and treatment characteristics

The mean target volume was 21.5 cm^3^ (range 7.37‐59.82 cm^3^). The mean MU was 2592 (range 1566–4505). The mean total delivery time was 4.44 min (range 2.67‐7.52 min) for all arcs (see Table 1). In general, all patients tolerated the CMB immobilization system extremely well and completed all five treatments (20‐30 min each) without interruption or significant delays because of discomfort.

### B. Interfractional setup errors

The initial setup errors were −0.8±1.3 mm, −0.8±1.6 mm, and 0.3±1.9 mm in AP, CC, and LR directions, respectively, and $0.1^{\circ }\pm 0.9^{{}^{\circ}}$, $-0.1^{\circ }\pm 0.7^{\circ }$, and $-0.1^{\circ }\pm 0.4^{\circ }$ for yaw, roll, and pitch, respectively. Eighteen out of 21 patients had at least 2 fractions requiring absolute shifts exceeding 2.0 mm. The mean 3D vector for all patients was 2.7±1.4 mm (range: 1.7‐4.6 mm). The corresponding PTV margins $m_{\text{PTV}(90,95)}$ without daily image guidance were 3.2 mm, 3.7 mm, and 5.0 mm in AP, CC, and LR directions, respectively. The initial rotational errors in yaw, roll, and pitch were 2.2°, 1.7°, and 2.4°, respectively.

### C. Residual errors (pretreatment, postcorrection)

All setup errors were corrected by ExacTrac X‐ray to within 1.0 mm and 0.6° in all directions using the robotic couch. Verification X‐rays showed residual errors of 0.0±0.2 mm, 0.0±0.3 mm, and 0.0±0.3 mm in the AP, CC, and LR direction and $0.0^{\circ }\pm 0.2^{{}^{\circ}}$, $0.0^{\circ }\pm 0.2^{{}^{\circ}}$, and $0.2^{\circ }\pm 0.2^{{}^{\circ}}$ in the yaw, roll, and pitch directions, respectively.

### D. CBCT and ExacTrac X‐ray agreement

Table 2 shows the difference between CBCT registration and ExacTrac verification X‐ray alignment results in both translational and rotational directions. The CBCT agreed with ExacTrac X‐ray results to <2 mm in all translational directions, and to $<2.5^{\circ }$ in all rotational directions. The 3D translational vector of this difference was 1.0±0.6 mm.

Group 1 (skull base targets) patients had significantly better agreement between CBCT and ExacTrac X‐ray with less physician reviewed adjustments compared to Group 2 (below C1 targets) patients. A disagreement >1.0 mm between CBCT and ExacTrac registration occurred in 50% of the treatments among Group 2 patients compared to 16.4% among Group 1 patients (p<0.001 for 3D Vector). The greatest disagreements occurred in the AP direction, with a maximum difference of 2.0 mm for Group 2 patients compared to 1.3 mm for Group 1 patients.

Fewer rotational discrepancies were also observed in Group 1 compared to Group 2 patients. While 16 out of 50 (32%) treatments from Group 2 exhibited a rotational discrepancy $>1.0^{\circ }$, only six out of 55 (11%) treatments from Group 1 exceeded 1.0° (p<0.01 in each direction). The calculated $m_{\text{PTV}(90,95)}$ for rotational discrepancies was 2.4° for Group 2 versus 1.3° for Group 1 (f<0.01).

After physician review of CBCT target volume, we found that >1 mm adjustments were performed in 2 of 55 (4%) treatments for Group 1 patients compared to 17 of 50 (34%) treatments for Group 2 patients (p<0.001 for 3D vector). The calculated PTV margins $m_{\text{PTV}(90,95)}$ without daily volumetric CBCT verification were 1.4 mm for Group 1 versus 2.9 mm for Group 2 (f<0.001).

**Table 2:** Agreement of CBCT and ExacTrac X‐ray alignments.

		*CBCT vs. ExacTrac Autoregistration*					
*Patient*		*AP (mm)*	*CC (mm)*	*LR (mm)*	*Yaw (°)*	*Roll (°)*	*Pitch (°)*
All Patient	Difference	0.0±0.8	−0.3±0.6	0.2±0.5	−0.4±0.8	0.1±0.5	0.3±0.7
	Σ	0.7	0.3	0.3	0.8	0.5	0.6
	σ	0.4	0.6	0.4	0.5	0.3	0.4
	$m_{\text{PTV}(90,95)}$	2.1	1.1	1.1	2.3	1.4	1.7
Group 1	Difference	0.2±0.5	−0.3±0.4	0.0±0.2	0.1±0.5	0.1±0.2	0.3±0.5
	Σ	0.5	0.3	0.2	0.4	0.2	0.4
	σ	0.2	0.3	0.2	0.3	0.2	0.2
	$m_{\text{PTV}(90,95)}$	1.4	0.9	0.6	1.3	0.7	1.2
Group 2	Difference	−0.2±1.0	−0.3±0.8	0.4±0.6	−0.9±0.9	−0.1±0.7	0.3±0.9
	Σ	1.0	0.3	0.3	0.8	0.7	0.8
	σ	0.5	0.8	0.5	0.6	0.4	0.6
	$m_{\text{PTV}(90,95)}$	2.9	1.4	1.2	2.4	2.0	2.3

Σ=systematic error; σ=random error.

### E. Intrafractional positioning errors

Table 3 lists the intrafractional positioning errors defined by prebeam ExacTrac X‐ray alignments. Each patient had at least two prebeam X‐ray alignments per treatment. All shifts detected were within 2.0 mm in each direction. The intrafractional rotational errors for both groups were $<2.0^{\circ }$. The 3D translational vector among all patients was 0.9±0.6 mm(range: 0.1‐3.0 mm). The vectors before beams 1, 2, and 3 were 0.9±0.6, 1.0±0.6, and 0.9±0.4, respectively. A 3D intrafractional difference <1.0 mm occurred in 83% of patients from Group 1 versus 50% of those from Group 2 (p<0.0005 for 3D vector). The recommended minimum uniform margin to account for intrafractional uncertainty was 1.5 mm for Group 1 compared to 2.0 mm for Group 2 patients.

**Table 3:** Intrafractional positioning errors.

*Patient*		*AP (mm)*	*CC (mm)*	*LR (mm)*	*Yaw (°)*	*Roll (°)*	*Pitch (°)*
All Patient	Errors	−0.1±0.6	−0.1±0.6	−0.2±0.5	0.0±0.5	0.0±0.6	−0.1±0.4
	Σ	0.3	0.5	0.4	0.4	0.5	0.2
	σ	0.6	0.5	0.5	0.4	0.4	0.4
	$m_{\text{PTV}(90,95)}$	1.1	1.6	1.4	1.3	1.5	0.8
Group 1	Errors	−0.1±0.4	−0.3±0.5	−0.1±0.4	−0.1±0.5	−0.1±0.3	−0.2±0.4
	Σ	0.3	0.4	0.4	0.4	0.3	0.3
	σ	0.3	0.4	0.3	0.3	0.2	0.3
	$m_{\text{PTV}(90,95)}$	0.9	1.3	1.3	1.3	1.0	0.9
Group 2	Errors	0.0±0.8	0.1±0.7	−0.4±0.6	0.0±0.6	0.0±0.8	0.0±0.5
	Σ	0.3	0.5	0.3	0.5	0.7	0.1
	σ	0.8	0.5	0.6	0.4	0.5	0.5
	$m_{\text{PTV}(90,95)}$	1.3	1.6	1.2	1.5	2.1	0.7

$SM = systematic error; σ = random error.

### F. Recommended PTV margin

Table 4 lists the minimal PTV margins required to achieve adequate coverage with a confidence level between 80% and 99% in each translational direction. Residual setup errors can be considered by adding 0.1–0.2 mm to the calculated margin.

**Table 4:** PTV margin (mm).

		*Confidence Level (%)*														
		*AP (mm)*					*CC (mm)*					*LR (mm)*				
*Patient*	*Dose Level*	*80*	*85*	*90*	*95*	*99*	*80*	*85*	*90*	*95*	*99*	*80*	*85*	*90*	*95*	*99*
All Patients	80%	0.8	0.8	0.9	1.0	1.1	1.3	1.3	1.4	1.6	1.9	1.1	1.1	1.2	1.3	1.6
	85%	0.9	0.9	1.0	1.0	1.2	1.3	1.4	1.5	1.6	1.9	1.1	1.2	1.3	1.4	1.6
	90%	0.9	1.0	1.0	1.1	1.2	1.4	1.4	1.5	1.7	1.9	1.2	1.2	1.3	1.4	1.7
	95%	1.0	1.0	1.1	1.2	1.3	1.4	1.5	1.6	1.7	2.0	1.2	1.3	1.4	1.5	1.7
	99%	1.1	1.2	1.2	1.3	1.5	1.5	1.6	1.7	1.8	2.1	1.3	1.4	1.5	1.6	1.8
Group 1	80%	0.7	0.7	0.8	0.9	1.0	1.0	1.1	1.1	1.3	1.5	1.0	1.1	1.2	1.3	1.5
	85%	0.7	0.8	0.8	0.9	1.0	1.1	1.1	1.2	1.3	1.5	1.1	1.1	1.2	1.3	1.6
	90%	0.8	0.8	0.9	0.9	1.1	1.1	1.2	1.2	1.3	1.6	1.1	1.1	1.2	1.4	1.6
	95%	0.8	0.8	0.9	1.0	1.1	1.1	1.2	1.3	1.4	1.6	1.1	1.2	1.3	1.4	1.6
	99%	0.9	0.9	1.0	1.0	1.2	1.2	1.3	1.4	1.5	1.7	1.2	1.2	1.3	1.5	1.7
Group 2	80%	0.9	1.0	1.0	1.1	1.3	1.3	1.3	1.4	1.6	1.9	0.9	1.0	1.0	1.1	1.3
	85%	1.0	1.1	1.1	1.2	1.4	1.3	1.4	1.5	1.6	1.9	1.0	1.0	1.1	1.2	1.4
	90%	1.1	1.1	1.2	1.3	1.4	1.4	1.5	1.5	1.7	2.0	1.1	1.1	1.2	1.3	1.4
	95%	1.2	1.2	1.3	1.4	1.5	1.4	1.5	1.6	1.7	2.0	1.1	1.2	1.2	1.3	1.5
	99%	1.4	1.4	1.5	1.6	1.7	1.6	1.6	1.7	1.9	2.2	1.3	1.3	1.4	1.5	1.7

## IV. DISCUSSION

In this study, we demonstrated that our customized CMB device highly restricted both translational and rotational head movement and, when used in combination with image guidance for online setup correction, provided robust immobilization to ensure minimum dose of 95% to target volume with 2.0 mm PTV margin for HN‐SABR.

In addition, we found that although IR‐based guidance can allow for faster setup times, the IR balls can underestimate the magnitude of the motion and rotation of the patient and are insufficient to provide enough setup accuracy because they are external markers on the mask and not directly on the patient's skin. We also noticed that the IR camera in treatment room was at times unable to position a few of the IR reflectors on the H&N masks during simulation, which may have increased our initial setup error results. This issue was addressed by repositioning of one or more balls on the first day of treatment to enable the system to start tracking. We would not expect this to affect our intrafractional setup uncertainty since alignment was performed using bony landmarks via the ExacTrac X‐ray and not with the IR markers. The IR markers are simply external fiducials utilized to ensure that the ExacTrac and CBCT based shifts were tracked to the correct position and accepted by the software system. Our verification ExacTrac X‐rays showed that the initial setup errors were corrected to within 1.0 mm and 0.6° for our patients.

We compared our results with recent publications in Table 5. Guckenberger et al.^(3)^ utilized CBCT and Elekta XVI software to measure interfractional positioning errors, whereas Tryggestad et al.^(5)^ utilized daily pre‐ and post‐treatment CBCT for these measurements. In Tryggestad's study, we listed the data obtained using their #4 immobilization device, which was their most robust immobilization system and utilized a similar three‐point customized CMB strategy as ours. Our systematic (or mean) and random (or standard deviation) translational setup errors were comparable to these reported studies.

Our intrafractional positioning errors compared favorably and were overall smaller than published studies, including that of Nakata et al.^(8)^ which investigated intrafractional setup reproducibility using ExacTrac X‐ray 6D IGRT system. Our measured random errors of 0.5–0.6 mm were around the order of the random error of the ExacTrac system, indicating that our CMB system greatly restricted target motion. Verbakel et al.^(14)^ used a phantom study and demonstrated that the accuracy of the ExacTrac positioning is approximately 0.3 mm (1 SD) in each direction. Jin et al.^(15)^ evaluated the “6D‐fusion” system of ExacTrac for target localization using an anthropomorphic head phantom and found the maximal random error of this system was 0.6 mm in each direction with 95% confidence interval.

**Table 5:** Comparison of setup errors among studies.

		*AP (mm)*	*CC (mm)*	*LR (mm)*	*Vector (mm)* (mean±SD) ^a^	*Yaw (°)*	*Roll (°)*	*Pitch (°)*
Interfractional Setup Errors	Guckenberger et al.^(3)^	0.7(1.2)	0.9(1.9)	0.8(1.4)		1.1(1.7)	1.1(1.4)	0.7(1.5)
	Tryggestad et al.^(5)^	0.9(0.8)	1.2(1.0)	0.7(0.9)	2.1±1.0	0.9(0.7)	0.8(0.8)	0.9(0.6)
	Our study (pre‐correction)	1.0(1.0)	1.1(1.3)	1.7(1.2)	2.7±1.4	0.7(0.6)	0.6(0.5)	0.8(0.4)
	Our study (residual setup error)	0.2(0.3)	0.2(0.3)	0.2(0.3)	0.4±0.3	0.1(0.2)	0.1(0.2)	0.2(0.3)
Intrafractional Setup Errors	Nakata et al.^(8)^	1.2(2.0)	0.6(0.6)	0.4(1.1)		0.1(0.8)	0.4(1.0)	1.5(1.0)
	Our study	0.3(0.6)	0.5(0.5)	0.4(0.5)		0.4(0.4)	0.5(0.4)	0.2(0.4)
	Tryggestad et al.^(5)^ ^a^	−0.1±0.4	−0.3±0.6	0.0±0.3	0.7±0.8	−0.1±0.6 −0.2±0.5	−0.1±0.4
	Our study^a^	−0.1±0.6	−0.1±0.6	−0.2±0.5	0.9±0.6	0.0±0.5	0.0±0.6	−0.1±0.4

a Results are shown in format of mean ± SD for all patients; otherwise, results are in format of Σ(σ).

SD=standard deviation; Σ=systematic error; σ=systematic error.

It deserves special mention that our rotational errors (systematic and mean) outperformed most published studies, as shown in Table 5. In particular, the pitch angle (chin movement) in our study appeared very robust. This may be clinically relevant as several studies have shown that pitch errors can exert the most distortion on head and neck axial imaging anatomy, thereby influencing target delineation and image registration.^16^, ^17^ Our Klarity AccuCushion is shaped to patient head, neck, and shoulder to create a secure custom mold. This appears to increase patient comfort and decrease the tension uncertainties of the neck during daily setup. Further restriction to head movement is reinforced with the addition of the bite‐block, which creates another index point by fixating the hard palate to the mask.

In general, our setup errors for HN‐SABR using ExacTrac X‐ray alignment and CBCT registration resulted in <2.0 mm and $<2.5^{\circ }$ agreement. Although both translational and rotational differences in Table 2 were evaluable from the volumetric matching process, only translational differences on CBCT (if >1 mm) were correctable due to limitations of the machine. Based on our CBCT data, we did observe variations in uncertainties for different head and neck treatment sites. For example, tongue base or oral cavity targets may see up to 3 mm 3D shifts, while targets closer to skull base and more centrally located, including retropharyngeal nodes, typically require <1.5 mm shifts and $<1.5^{\circ }$ rotations. This observed increase in uncertainty for targets further from the skull base is consistent with reports from Zhang et al, and other studies.^11^, ^18^ For these patients, bony alignment on 2D images may be suboptimal. Although cervical spine curvature and jaw position are significantly minimized with our CMB setup, uncertainties due to internal organ motion and changes in soft‐tissue volume during the course of treatment for the head and neck region require further investigation.

PTV margins were calculated from initial (interfractional) setup errors, intrafractional positioning uncertainties, and CBCT detected uncertainties. Based on our initial setup errors, a 5.0 mm margin is recommended to account for target uncertainties when using our CMB immobilization in the absence of daily Exactrac X‐ray (or any IGRT verification). In our SBRT practice, we utilize both ExacTrac X‐ray and CBCT image guidance, thereby minimizing the required PTV margin for SBRT. Since these initial setup errors and CBCT detected errors are corrected prior to each treatment, our primary concern during SBRT is intrafractional uncertainties from patient and target motion during the treatment. Our results showed up to 1.3 mm and 1.6 mm intrafractional uncertainty for Group 1 and 2 patients, respectively, with a 2.0 mm PTV margin ensuring target minimum dose of 95%. Our CBCT data indicate that, if the Exactrac X‐ray is used alone without volumetric CBCT (or soft‐tissue image guidance), a 1.4 mm margin is needed to account for uncertainty of skull base targets, whereas a 2.9 mm margin is needed to account for uncertainty of movable targets below C1.

We listed PTV margins for different confidence level in Table 4, based on the systematic and random errors measured in our study, to guide margin determination for tumor coverage. Our setup with daily ExacTrac X‐ray and CBCT image guidance showed target mininum dose of 99% when using a target volume to PTV margin of <2 mm to achieve a 95% confidence level. With additional considerations of mechanical characteristics and limitation of treatment machine and coincidence of imaging system and radiation delivery system, our current clinical margins of 2–3 mm were reasonable. For tumors located in areas with higher uncertainty, such as in the tongue base or oral cavity, or more inferiorly along the cervical spine, a 3 mm margin was typically used.

## V. CONCLUSIONS

A robust CMB immobilization device was used for HN‐SABR reirradiation patients to greatly restrict patient motion during treatment. Our results appear to outperform published studies in both translational and rotational positioning errors. With image guidance for online setup correction, a PTV margin of 2.0 mm appears adequate for our HN‐SABR patients. Three‐dimensional alignment using target volume is recommended to account for daily setup uncertainties in regions with possible oral motion and/or flexibility of spine column.

## COPYRIGHT

This work is licensed under a Creative Commons Attribution 4.0 International License.
